# UHPLC-MS-based metabolomics and chemoinformatics study reveals the neuroprotective effect and chemical characteristic in Parkinson’s disease mice after oral administration of Wen-Shen-Yang-Gan decoction

**DOI:** 10.18632/aging.203361

**Published:** 2021-08-02

**Authors:** Guoxue Zhu, Wang Wang, Chang Chen, Lili Tang, Yan Liang, Zhennian Zhang, Yan Lu, Yang Zhao

**Affiliations:** 1 Department of Neurology, Nanjing Hospital of Chinese Medicine Affiliated to Nanjing University of Chinese Medicine, Nanjing University of Chinese Medicine, Nanjing, Jiangsu, China; 2 School of Medicine and Holistic Integrative Medicine, Nanjing University of Chinese Medicine, Nanjing, Jiangsu, China; 3 Chinese Medicine Modernization and Big Data Research Center, Nanjing Hospital of Chinese Medicine Affiliated to Nanjing University of Chinese Medicine, Nanjing University of Chinese Medicine, Nanjing, Jiangsu, China; 4 Medical School of Nanjing University, Nanjing, Jiangsu, China

**Keywords:** Parkinson's disease, Wen-Shen-Yang-Gan decoction, DA neurons apoptosis, UPLC-Q-TOF-MS, multivariate statistical analysis

## Abstract

Parkinson’s disease (PD), the typical neurodegenerative disease, is characterized by the progressive loss of dopaminergic neurons in the substantia nigra (SN). However, no therapeutic agent used currently could slow down neuronal cell loss so as to decelerate or halt the progression of PD. Traditional Chinese medicine (TCM) has been utilized to treat the dysfunction of the autonomic nervous system. Wen-Shen-Yang-Gan decoction (WSYGD) has a good effect on the clinical treatment of PD with constipation. However, it is not clear which ingredients and what mechanism are responsible for the therapeutic effect. In this study, the pharmacodynamic study of WSYGD in PD mice was applied. Concurrently, a novel method for the identification of metabolic profiles of WSYGD has been developed. Finally, we found that WSYGD could protect the PD mice induced by rotenone. The underlying mechanism of the protective effect may be related to the reduction of the DA neurons apoptosis via reducing inflammatory reaction. By virtue of UPLC-MS and chemoinformatics method, 35 prototype compounds and 27 metabolites were filtered out and tentatively characterized. In conclusion, this study provides an insight into the metabolism of WSYGD *in vivo* to enable understanding of the metabolic process and therapeutic mechanism of PD.

## INTRODUCTION

Parkinson's disease (PD) is the second ranked primary neurodegenerative disease of central nervous system with dysfunction in cortices which affects 2-3% of the aging population [1, 2]. Patients typically manifest with motor manifestations, such as movement retardation, stiffness, rest tremor, and postural instability, and non-motor features [3]. Although, the gold standard tablet treatment for motor symptoms of PD is still levodopa while other types of drugs such as MAO-B inhibitors or beta-blockers have also been utilized [4]. However, none of the therapeutic agents in previously could slow down the loss of neuronal cells so as to decelerate or halt the progression of PD [4, 5]. Levodopa-resistant symptoms even conduce to disability and significantly increase the risk of all-cause mortality [6]. Thereby, it is crucial and urgent to search appropriate therapeutic agents with better treatments for PD with western medicine.

Wen-Shen-Yang-Gan decoction (WSYGD) is a TCM utilized for PD by the Nanjing Hospital of Traditional Chinese Medicine, it is formulated by Professor Yang Zhao according to the pathogenesis of endogenous liver wind agitation. It is effective to treat PD with constipation [7, 8] and composed of six Chinese herbs: Cistanches Herba (Roucongrong in China, RCR), Paeoniae Radix Alba (Biaoshao in China, BS), Linderae Radix (Wuyao in China, WY), Alpiniae Oxyphyllae Fructus (Yizhi in China, YZ), Dioscoreae Rhizoma (Shanyao in China, SY), Uncariae Ramulus Cum Uncis (Gouteng in China, GT). Our preliminary study showed that the Wen-Shen-Yang-Gan decoction has protective effects on the PD patients and rotenone subacute model mice [9, 10]. However, compared with studies of its clinical application, the fundamental research of WSYGD alleviates PD have been inadequate.

The metabolism study of TCM will be of vital importance in the discovery and development process of new drugs [11]. Therefore, the research of metabolites and metabolomics-guided drug metabolism allow researchers to investigate the biomarker-clinical relevance [12]. In this respect, the identification of disease biomarkers in metabolomics provides distinctive views on the causes of disease [13, 14]. High performance liquid chromatography coupled with tandem mass spectrometry (LC-MS/MS) have been widely utilized due to, high selectivity, increased sensitivity and high chromatographic resolution, in complex biological matrices [15]. Multivariate statistics, such as principal component analysis (PCA) and orthogonal partial least squares discriminant analysis (OPLS-DA), could be solve the problem that small differences between highly similar species might not be tested but can largely impact the health of outpatients. In the present study, (1) behavioral tests (pole test and rotarod test), were utilized to evaluate the motor and behavioral performance of PD mice in the different groups; (2) the tyrosine hydroxylase (TH) immunofluorescence staining was employed to detect the protection of WSYGD on the damage in striatal neurons; (3) ELISA was utilized to detect the inflammatory cytokines in mice’s serum; (4) UPLC-Q-TOF MS/MS combined with chemoinformatics (PCA and OPLS-DA) for the characterization of metabolic profiles in mouse plasma after oral administration of WSYGD is developed and fully validated to screen out the ingredients absorbed into blood.

## MATERIALS AND METHODS

### Chemicals and reagents

Acetonitrile and methanol (HPLC grade, >=99.9%) were supplied by Merck ltd. Formic acid was obtained from Sigma-Aldrich. Ultra pure water was purified using the Milli-Q system (Bedford, USA). The reference compounds of rhynchophylline (lot number: wkq18051803), verbascoside (lot number: 120524), linderane (lot number: wkq18031303), geniposidic acid (lot number: wkq18040312), coclaurine (lot number: wkq18036198) were supplied by Sichuan Weikeqi- Biotech Co., Ltd., nootkatone were obtained from Chengdu Chroma- Biotechnology Co., Ltd., rutin (lot number: 100080-201811), echinacoside (lot number: 111670-201907), quercetin (lot number:081-9003), and paeoniflorin (lot number:110736-201943) were obtained from National Institutes for Food and Drug Control.

### Plant materials

Cistanches Herba, Alpiniae Oxyphyllae Fructus, Dioscoreae Rhizoma, Paeoniae Radix Alba, Linderae Radix and Uncariae Ramulus Cum Uncis were obtained from Zhengzhou Ruilong pharmaceutical CO., Ltd. and authenticated by Professor Shihui Qian (Jiansu Province Academy of Traditional Chinese Medicine, Nanjing, China).

### Water extract of WSYGD preparation

The weighed powders of RCR (30g), BS (30g), WY (20g), YZ (30g), SY (20g), GT (20g) were mixed and immersed in 1500 mL of deionized water for 30 min then decocted using boiling for 1 h. The procedure was repeated twice. The extract was decanted and evaporated under reduced pressure. The mixed decoction was concentrated to high dosage (4g/mL), medium dosage (2g/mL), low dosage (1g/mL). The total ion chromatogram of WSYGD extract of medium dosage were shown in Supplementary Figure 1.

### Animal treatment and sample preparation

### Animals and treatments

Male C57BL/6J mice (ten months old, weighing 30-35g) were provided by Shanghai SLAC Laboratory Animal Co., Ltd (License No. SCXK (Hu), 2007-0005). Animals were housed in a controlled condition (22±2° C, 55±5% humidity) with a 12h circadian rhythm and had free access to food and water for two weeks to acclimatize them to the environment. Then, they were divided into control group, model group, Wen-Shen-Yang-Gan decoction groups (WSYGD-H, WSYGD-M and WSYGD-L) and drug group (Sinemet, carbidopa/levodopa, 25/100mg. 50mg/kg) randomly, 10 mice in each group. The mice in the constituent research divided into control group and model group, 6 mice in each group. All protocols and care for the mice were performed in accordance with the Guidelines for the Care and Use of Laboratory Animals and approved by the Animal Ethics Committee of Nanjing University of Chinese Medicine.

### Efficacy of WSYGD to Parkinson’s disease

**Figure 1:**
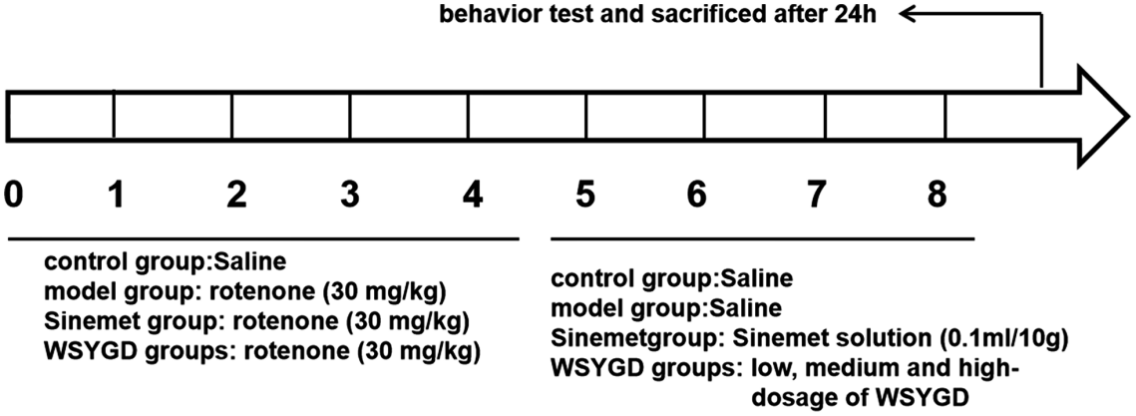
Schematic representation of experiment.

All of the mice except for control group were intragastrically administered with rotenone (30 mg/kg, lot number: R8875, sigma, USA). For the control group, an equal volume of normal saline was treated once daily for four weeks. In the 5th to 8th week, the administrated groups mice were received WSYGD solution once a day with different dose (4g/mL, 2g/mL, 1g/mL) and the positive group were received Sinemet solution (0.1ml/10g, MSD pharmaceutical Co. Ltd.). The mice of other two groups were given an equal volume of normal saline. The Behavioral of mice were evaluated by pole test and rotarod test at 24 h after experiment. All the animals were sacrificed 24h after the behavior test. The concrete drug regimen shown in Figure 1.

### Constituents analysis of WSYGD

The male mice of C57BL/6J were divided into Control group and control-WSYGD group. The control group intragastrically administrated with normal saline each day. The mice of control-WSYGD group received WSYGD extract (dissolved in normal saline) at 14.70 g**/**kg. All animals were anesthetized with 10% urethane. The blood was obtained from the fosse orbital vein of animals at 0.25, 0.5, 1, 2, 3, 4, 6, 8 h after WSYGD treatment.

The blood samples (with heparin and without anticoagulant) were centrifuged (12000 rpm, 10 min, 4° C) to acquire plasma. The sample of various points from one animal were mixed and stored at -80° C until analysis.

### Pole test

The pole test was performed on all mice to assess movement retardation and coordination. A steel pipe (50 cm long and 1 cm in diameter) which is tightly wrapped by white antiskid belt and has a spherical protrusion (2 cm in diameter) on the top as attachment points of mice. Place the mouse’s head upward on the spherical protrusion, record the time from its placement on the pole top to the point of turning its head downwards and the total time from its placement on the pole top to its climbing to the pole bottom with its rear limbs touching the ground. Three trials were performed with each mouse with an interval of two minutes and median data were taken across the trials.

### Rotarod test

The rotarod test was performed with the help of ZH-600 mouse rotarod fatigue meter (Anhui Zhenghua biologic apparatus facilities Co., Ltd). The mice were placed on a rotation drum and measurement the time of standing on the drum as the speed accelerated from five to forty rpm in five-minute. Place the mice on a shifting drum, increase the rotation speed from 5 rpm to 40 rpm in 5 minutes, and record the time of standing on the drum. Final trials were performed three times for each animal in every group, and the values were averaged for every animal. The experimental environment, light and temperature for example, and timing were compatible in all the tests.

### Immunofluorescence staining

After perfusion, the tissue samples of mice were rapidly removed and fixed with 4% paraformaldehyde overnight. Sucrose solution was utilized to store isolated brains to dehydrate gradiently and embedded in OCT for frozen tissue sections. The substantia nigra brain tissue sections in line with the brain atlas were prepared. Goat anti-mouse IgG (H&L, ab150113, Abcam, Cambridge, UK) was appended and hatched for one hour after being flushed with PBS. The sample was washed by PBS and sealed with anti- fluorescence. The flake was observed by Olympus BX63 fluorescent microscope (Olympus Corporation, Tokyo, Japan).

### Western blotting (WB) analysis

Mid-brains of mice were rinsed with PBS and lysed with radioimmunoprecipitation assay buffer and then centrifuged (12000 rpm, 10 minutes, 4° C) Then, Lysates were segregated by 12% SDS-PAGE and transferred to polyvinylidene fluoride membrane (Millipore, Danvers, MA, USA), blocked in 5% BSA in PBS and pre-incubated with primary antibodies as the α-syn (ab59264, Abcam, Cambridge, UK), TH (SAB4200697, Sigma-Aldrich, USA) and GAPDH (60004-1-Ig, ProteinTech Group, Inc., USA). The GAPDH antibody was utilized as a loading control. On the second day, the PVDF membrane was incubated after flushed by TBST solution for one hour at 4° C. The membranes were exposed to anti-rabbit IgG antibodies (HRP, ZB-2301, Beijing Zhongshan Jinqiao Biotechnology Co., Ltd., China) for one hour at 4° C and scanned with a chemiluminescence image analysis system (Tanon-5200, Tanon Science and Technology Co., Ltd, China).

### Quantization of cytokine levels by enzyme-linked immunosorbent assay (ELISA)

The blood was collected and clarified, then centrifugated at 12000 rpm for 10 min at 4° C, and the supernatant was utilized for analysis. The levels of supernatant cytokines (all of obtained from Shanghai ZCi Biotechnology Co., Ltd., China), such as IL-10, TNF-α, and IL-1β, were determined by ELISA kits. Standards and samples were incubated by diluent in a plate coated with primary antibody for one hour at room temperature. Plates were then washed three times and 100 μL of substrate solution (tetramethylbenzidine) was added to each well for chromogenic reaction for 15 minutes in complete darkness. Diluted standards and samples were appended to each well and incubation with biotin-labelled secondary antibodies. Then, the plate was incubated by horseradish peroxidase-conjugated streptavidin (SA-HRP). The reaction halted by adding 50 μL of stop solution into each well. The standard curve was constructed using the absorbance of each standard (450nm, vertical axis) against its concentration (horizontal axis). The concentration of inflammatory cytokines was calculated using the relative absorbance of the samples and standards.

### Reference and plant sample preparation

A standard mixture containing rhynchophylline, verbascoside, linderane, geniposidic acid, coclaurine, nootkatone, rutin, echinacoside, quercetin and paeoniflorin was prepared in 100% methanol.

An aliquot of 10 milliliter of the WSYGD solution was decanted and evaporated under reduced pressure. The residue was dissolved in 10 milliliter of methanol and vortex-mixed for 5 min. The sample was centrifugated with 12000 rpm for 10 min at room temperature and then filtered through a 0.22 mm membrane.

### Instrument and analytical conditions

Metabolite analysis was achieved using a Waters ACQUITY UPLCTM I-Class chromatography system (Waters Corp, Milford, MA, USA) equipped with ion mobility mass spectrometry detector. The separation was performed on a Thermo Scientific™ Syncronis™ C18 (100 mm×2.1 mm, 1.7 mum particle size, Thermo Fisher Scientific) and the injection volume was 5 μL at 25° C. A gradient elution profile was employed using mobile phase A (0.1% formic acid in acetonitrile) and mobile phase B (0.1% formic acid in water). The following gradient was utilized: 0 min, 5% A; 0.5 min, 5% A; 3 min, 40% A; 10.5 min, 95% A. The flow rate was set to 0.40 mL/min.

The mass spectrometry was carried out using a SYNAPT G2-Si Q TOF (Waters Corp, Wilmslow, UK) connected to the ACQUITY UPLC I-Class System via an ESI interface. The mass spectrometry analysis was performed in the positive mode and negative mode, with the following parameters: Capillary 1.5 kV, sample voltage 30 V, cone voltages 5 V, source temperature 120° C, desolvation temperature 500° C. desolvation gas flow rate 800 L/h, cone gas flow rate 50 L/h. The MS was set with 0.1 second of scan time to acquire in sensitivity mode. The accurate mass precursor and fragment ion data was obtained using the selected mass range of m/z 100-1200.

### Data processing

### The mechanism of WSYGD in PD

All data were expressed as mean ±SD and statistical significance by using one-way ANOVA followed by post-hoc analysis using the GraphPad Prism software, (version 8.0, San Diego, CA, USA). Ultimately, the data that P < 0.05 was deemed to be significant.

### Composition identification

The resultant data matrices from the analytical technique were introduced into the Masslynx™ NT 4.1 software (Waters Corporation) for multi-variate statistical analysis including PCA and OPLS-DA. Pareto scaling was performed through Principal component analysis to catch sight of the separation between groups, and OPLS-DA to recognition important characteristics using Masslynx v.4.1 (Waters, Milford, MA, USA) [16]. We utilize the database of HMDB, Massbank and KEGG to screen and discriminate the potential markers.

## RESULTS AND DISCUSSION

### Effects of WSYGD on behavioral test of rotenone-intoxicated mice

**Figure 2:**
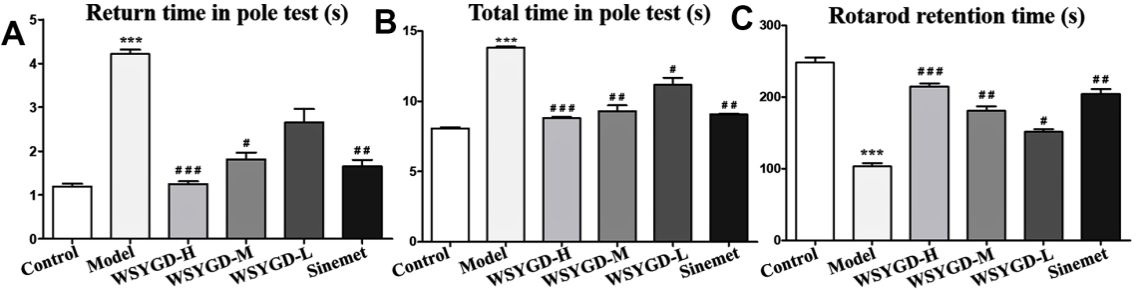
**Effects of WSYGD on behavioral test of PD chronic model mice.** In the pole test, (**A**) refers to the turning time and (**B**) refers to the total time; (**C**) refers to the retention time in the rotarod test. Control: blank group; Model: rotenone-intoxicated group; WSYGD-H: high dosage group; WSYGD-M: medium dosage group; WSYGD-L: Low dosage group; Sinemet: positive control. ***p<0.001 vs. Control; #p<0.05,##p<0.01, ###p<0.001 vs. Model.

In the behavioral test of pole, the turning around time of mice head in the rotenone-intoxicated group was obviously longer than control group mice, while the turning around time of mice head in the WSYGD-H, WSYGD-M and WSYGD-L groups were significantly shortened, indicating statistically significant in comparison with model. With regard to the total time of climbing, the mice in rotenone-intoxicated group obviously longer than those in control group, and the time spent by WSYGD-H and WSYGD-M was significantly shortened, indicating statistically significant in comparison with model. In behavioral test of rotarod, the time of rod-standing time of rotenone-intoxicated group mice were obviously shorter than that of control, moreover, the WSYGD-H, WSYGD-M and WSYGD-L group mice were obviously longer than rotenone-intoxicated group, the results shown in Figure 2.

### Effects of WSYGD on midbrain dopamine (DA) neurons and pathological changes of α-syn in rotenone-intoxicated mice

**Figure 3:**
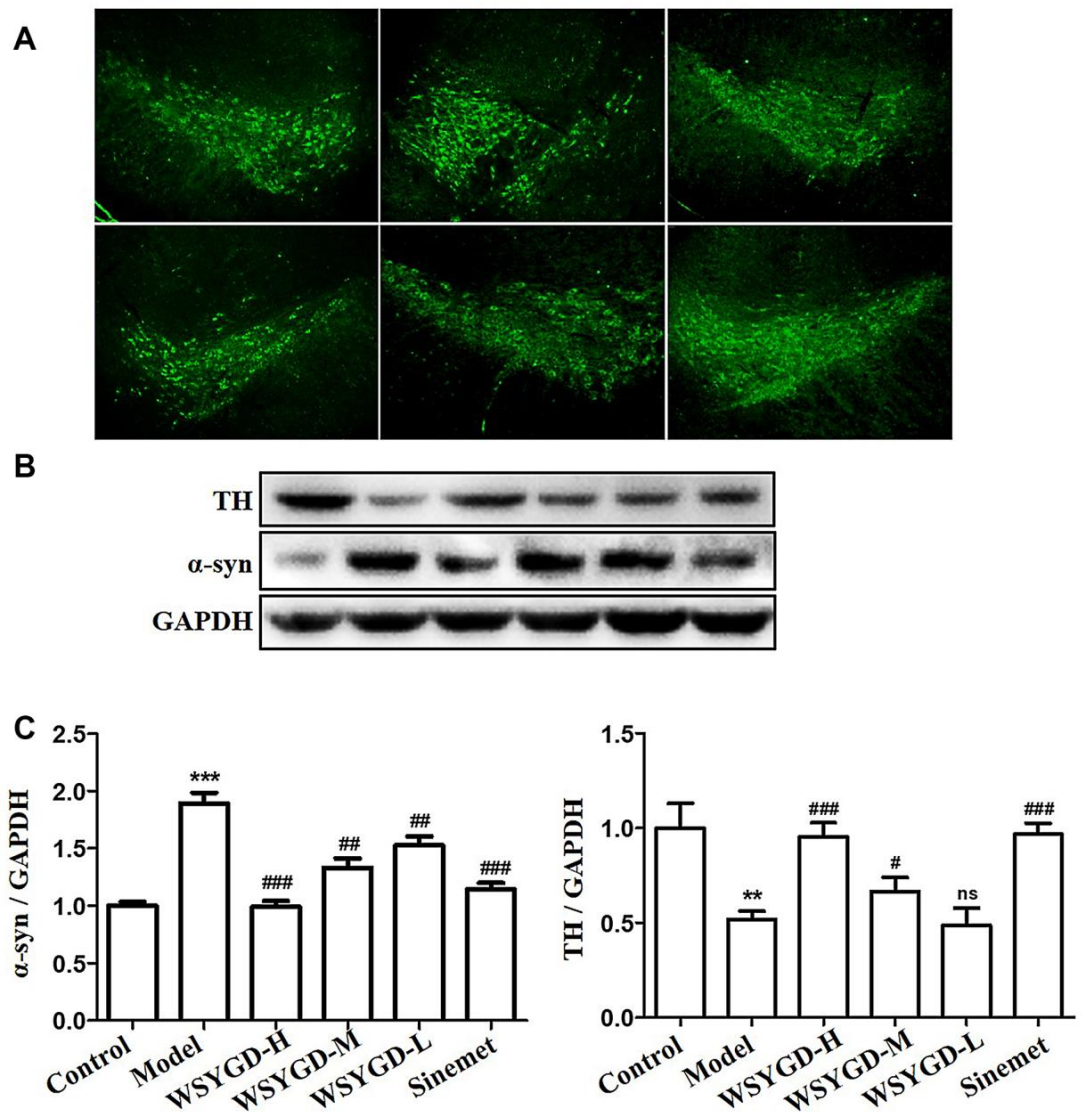
Immunofluorescence staining of TH protein in the substantia nigra of mice (**A**); Western blotting analysis (**B**) and quantification (**C**) of relative α-syn and TH protein abundance. Control: blank group; Model: rotenone-intoxicated group; WSYGD-H: high dosage group; WSYGD-M: medium dosage group; WSYGD-L: Low dosage group; Sinemet: positive control. **p<0.01,***p<0.001 vs control; #p<0.01, ##p<0.01, ###p<0.001 vs Model.

The TH-positive cells number could be utilized to reflect the dopamine neurons numbers. The WB results shown that the TH expression in rotenone-intoxicated group was evidently down-regulated than that in the control group, and that in WSYGD-H, WSYGD-M and WSYGD-L groups were significantly increased in comparison with that in model group (Figure 3B). The same results were also indicated among the immunofluorescence test (Figure 3A). Simultaneously, the α-syn expression among the middle brain of mice in the rotenone-intoxicated group was evidently upward-regulated than that in the control group, and WSYGD-H, WSYGD-M and WSYGD-L could obviously decrease the expression of α-syn (Figure 3B).

### Effects of WSYGD on peripheral immunity of rotenone-intoxicated mice

**Figure 4:**
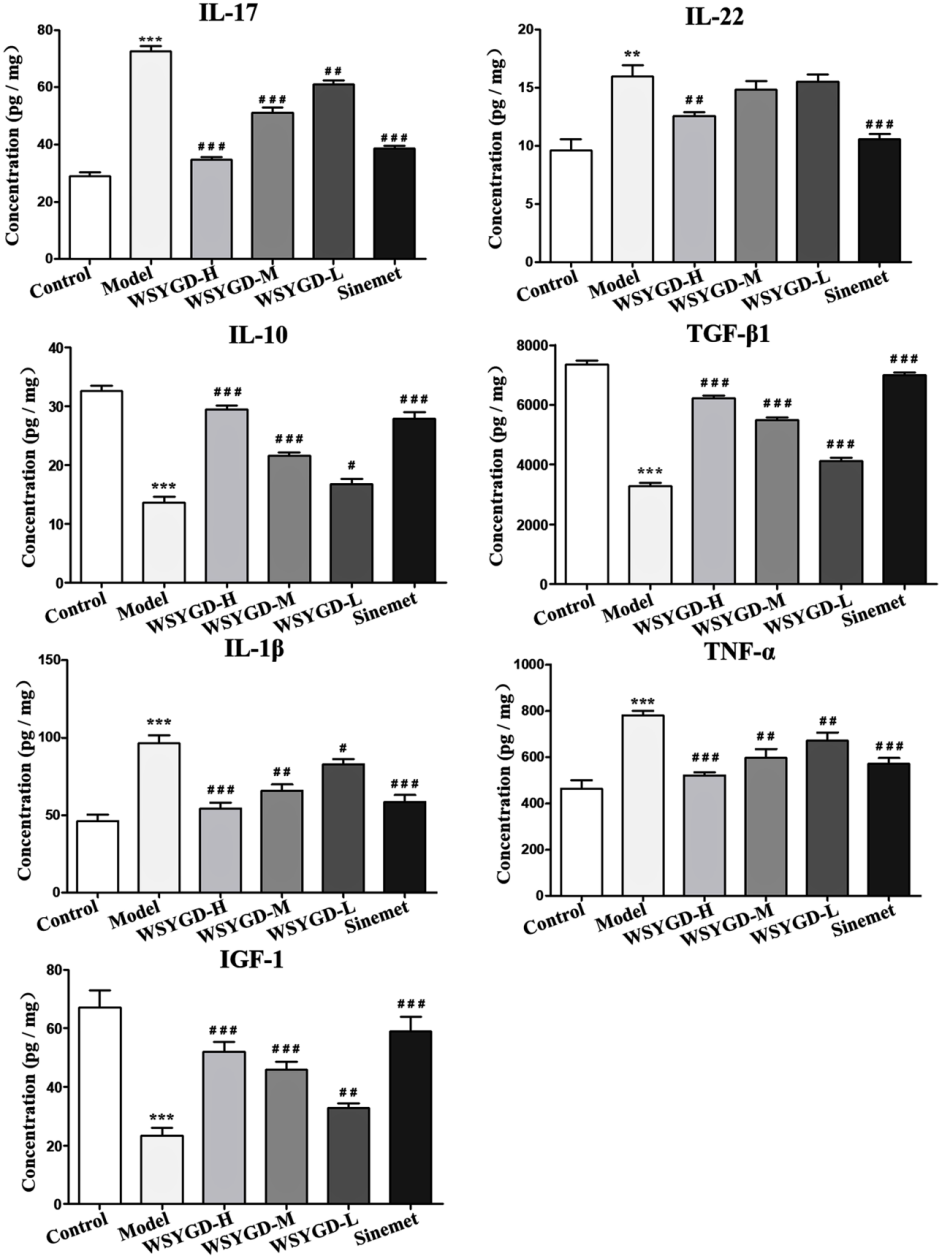
**Changes of serum inflammatory factors including IL-10, IL-17, IL-22, IL-10, TGF-β1, TNF-α, IGF-1, IL-1β and NF-κB in Mice.** Control: blank group; Model: rotenone-intoxicated group; WSYGD-H: high dosage group; WSYGD-M: medium dosage group; WSYGD-L: Low dosage group; Sinemet: positive control. **p<0.001, ***p<0.001 vs control; #p<0.05, ##p<0.01, ###p<0.001 vs Model.

The result of ELISA shown that the inflammatory cytokines contents such as TNF-α, IFN-γ, IL-1β, IL-22, and IL-17 in the mice serum of rotenone-intoxicated group were obviously increased than those in normal group. However, IGF-1 and TGF-β1 in rotenone-intoxicated group were evidently lower than that in the normal group. Simultaneously, WSYGD could reverse such phenomenon (Figure 4).

### UPLC-MS characterization of chemical components from WSYGD

**Figure 5:**
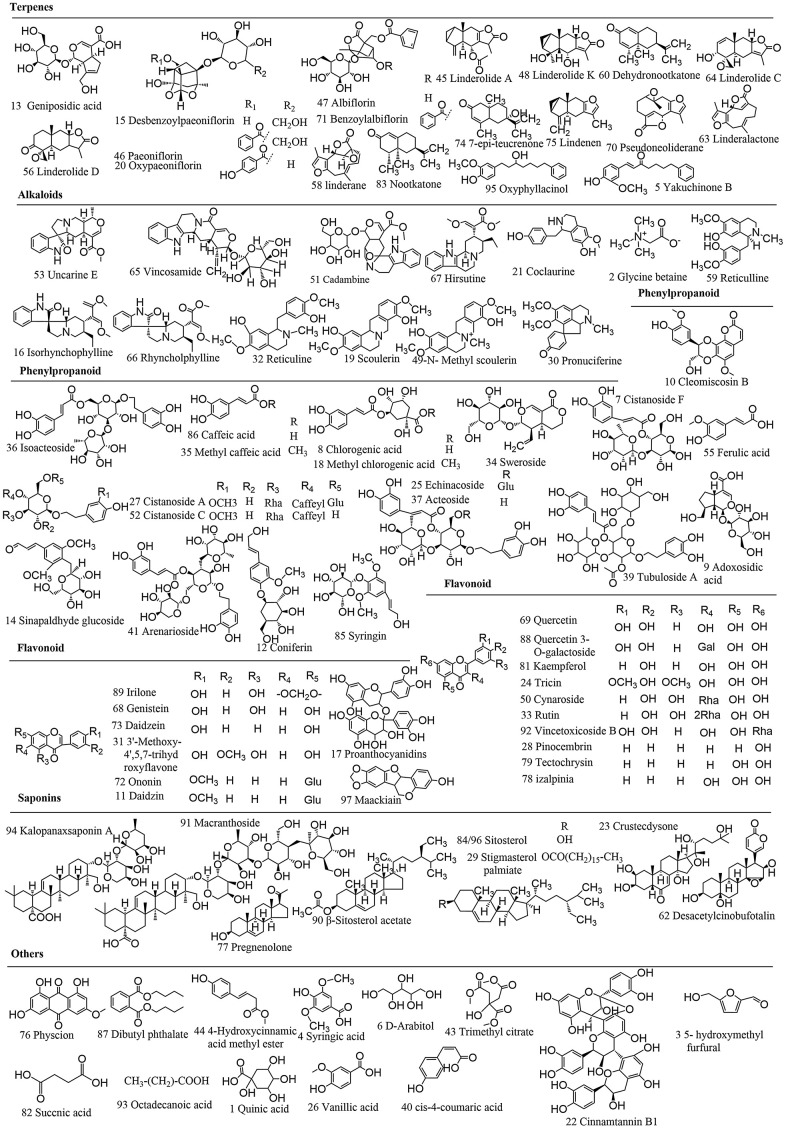
Chemical structures of WSYGD identified using UPLC-ESI-Q-TOF MS.

All mass spectrum data acquired in the LC-MS/MS analysis were utilized to recognize the metabolites structures which was essential for structural confirmation of the biomarkers by the procedures as described in the before-mentioned. The UPLC-QTOF/MS analysis platform provided the precise molecular mass within measurement errors (<5 ppm), allowing the potential element composition, unsaturation degree and fractional isotope abundance of the compounds to be determined. Total ion chromatograms were obtained by LC-MS/MS (Supplementary Figure 1). A total of 97 peaks were obtained and confirmed or tentatively preliminarily peculiarity (Figure 5 and Supplementary Table 1). For instance, the molecular weight of 37 peak is 623.1969, and the main fragment ions analyzed by MS/MS screening were surveyed at m/z 605.1870 [M-H_3_O]-, 487.1452 [M-C_8_H_9_O_2_]-,477.1397 [M-C_6_H_11_O_4_]-, 179.0344 [M-C_20_H_29_O_11_]-, 161.0239 [M-C_23_H_25_O_10_]-. Therefore, the elemental composition of ion 37 was calculated to be C_29_H_36_O_15_ and identified as acteoside.

### Multivariate statistical analysis for *in vivo* discrimination of WSYGD

**Figure 6:**
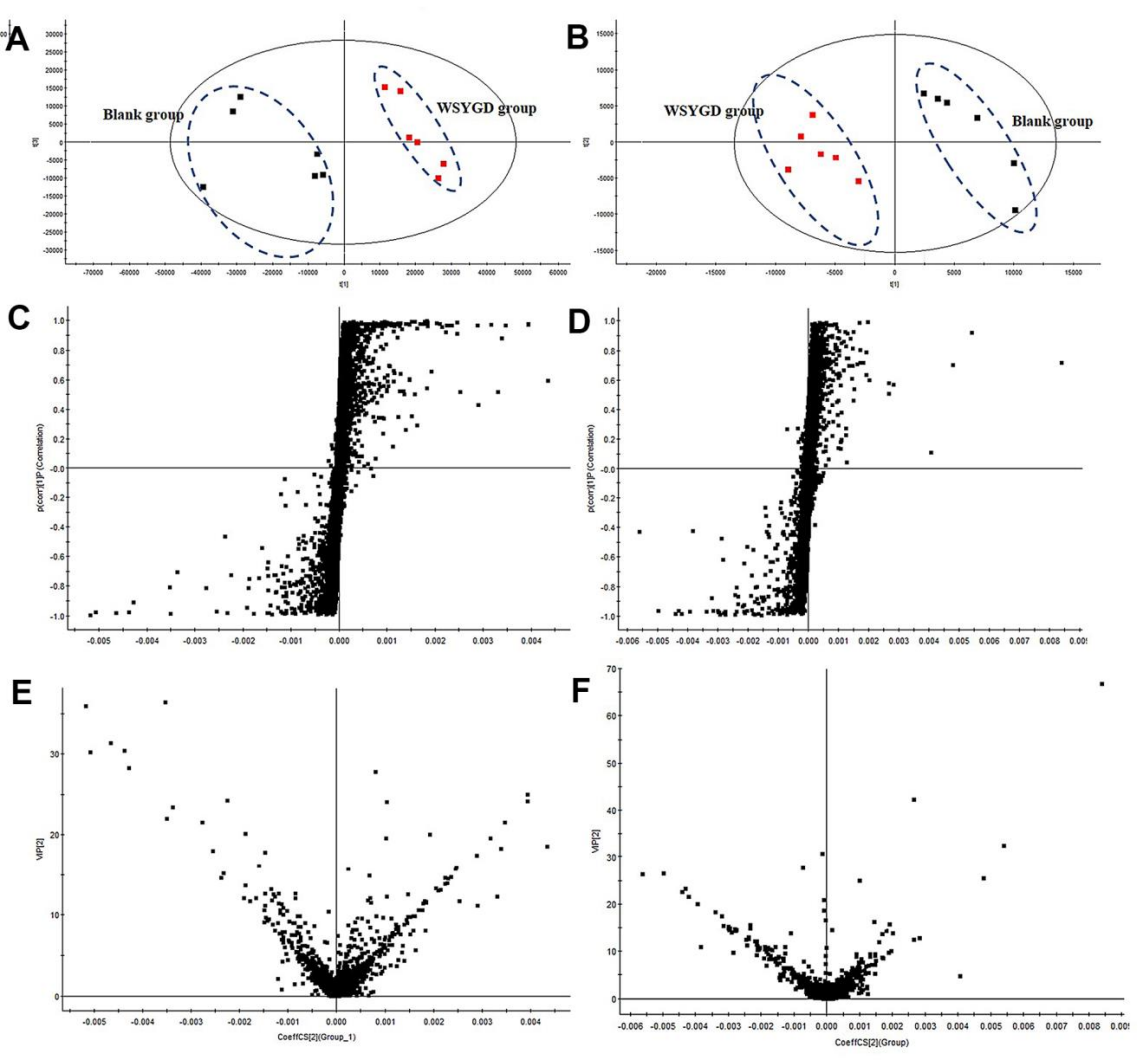
PCA score plot of all analyzed samples in positive-ion (**A**) mode and negative-ion (**B**) mode with the statistical parameters; S-plot of OPLS-DA in positive-ion (**C**) mode and negative-ion (**D**) mode with the statistical parameters; VIP value plot in positive-ion (**E**) mode and negative-ion (**F**) mode.

The raw data were collected by the SYNAPT G2-Si Q-TOF before analysis. The representative total ion chromatograms (TICs) of WSYGD in mice were shown in Supplementary Figure 2. To compare the difference between control group and WSYGD group, unsupervised PCA and supervised OPLS-DA were produced and the results data were displayed as PCA scores plot (Figure 6A, 6B). The samples from WSYGD and normal group clusters were obviously diverse indicating that the clear discrimination between two groups.

To find the potential quality markers, OPLS-DA analysis was performed to emerge S-plot (Figure 6C, 6D). As it could be observed in Figure 6C, 6D every point symbolizes an ion t_R_-m/z pair; the X-axis symbolizes the ingredient contribution and the Y-axis symbolizes the ingredient confidence. The farther away the distance is from the origin the greater the contribution to classification. Thus, the variables located at the two ends of “S” sharp were the compounds which are more important to discriminating the two groups, namely potential characteristic chemical markers. Then, to minimize complex data, further filtering procedures based on VIP value (VIP>1) analysis were performed (Figure 6E, 6F). It turns out that 62 variables were screened out, including 23 and 39 ions in positive mode and negative mode, respectively. Simultaneously, the result of the variables being summarized in ESI, Table 1.

**Table 1:** Identification of prototype compounds and metabolites of WSYGD in mouse plasma by UPLC-ESI-Q-TOF MS.

**No.**	**t_R_ (min)**	**Detected mass [M-H]^-^/[M+H]^+^**	**Theoretical exact mass (Da)**	**Mass error (ppm)**	**Molecular formula**	**MS/MS (m/z)**	**Assigned identity**	**Source**
M1	0.402	201.1120	201.1127	-3.5	C_10_H_16_O_4_	201.1120,157.1128	Paeonimetabolin II	PL
M2	0.720	297.0616	297.0610	2.0	C_13_H_14_O_8_	121.0132, 103.0170	Benzoic acid-O-glucuronide	PL
P1	0.771	285.0760	285.0763	-1.1	C_16_H_12_O_5_	285.0760, 307.0574, 323.0312	Maackiain	UR
M3	0.802	232.9749	232.9756	-3.0	C_7_H_6_O_7_S	153.0181	Protocatechuate-3-O-sulfate	PL
P2	0.803	118.0871	118.0868	2.5	C_5_H_11_NO_2_	118.0871, 74.0972	Glycine betaine	CH
P3	0.950	119.0344	119.0344	0	C_4_H_6_O_4_	119.0344, 141.0171, 136.0615	Succnic acid	AO
P4	1.330	181.0493	181.0501	-4.4	C_9_H_8_O_4_	163.0391, 151.0385, 135.0441, 105.0341, 71.0133	Caffeic acid	UR
P5	1.604	375.1320	375.1291	4.5	C_16_H_22_O_10_	375.1284, 313.1287, 213.0727, 179.0708, 121.0653	Geniposidic acid	CH
M4	1.614	279.0172	279.0175	-1.1	C_9_H_10_O_8_S	279.0172, 199.0604, 300.9994	3,4-di-methoxygallate sulfate	PL
P6	1.625	311.1649	311.1647	0.6	C_20_H_22_O_3_	311.1649	Yakuchinone B	AO
P7	2.020	371.1334	371.1342	-2.2	C_17_H_22_O_9_	371.1334, 185.0809	Sinapaldhyde glucoside	CH
P8	2.100	355.1015	355.1029	-3.9	C_16_H_18_O_9_	355.1015, 127.1123	Chlorogenic acid	UR
P9	2.239	415.1018	415.1029	-2.6	C_21_H_20_O_9_	415.1018, 399.1080	Daidzin	DO
P10	2.278	369.1172	369.1186	-3.8	C_17_H_20_O_9_	369.1172, 333.0968, 259.0818	Methyl chlorogenate	UR
P11	2.281	577.1325	577.1346	-3.6	C_30_H_26_O_12_	577.1325, 287.0614, 559.1246	Proanthocyanidins	LR
P12	2.290	343.1408	343.1393	4.4	C_16_H_22_O_8_	343.1408, 165.0922, 325.1274	Coniferin	CH
P13	2.340	314.1393	314.1392	-1	C_18_H_21_NO_4_	314.1393,298.1083, 296.1290,286.1445, 282.1133, 270.1135, 256.1348, 192.1022	Coclaurine	LR
P14	2.479	329.0654	329.0661	-2.1	C_17_H_14_O_7_	329.0654, 375.0728	Tricin	UR
P15	2.560	785.2506	785.2504	0.3	C_35_H_46_O_20_	623.2489, 461.2521, 315.2489, 161.2402	Echinacoside	CH
P16	2.610	235.0810	235.0818	-3.4	C_9_H_14_O_7_	235.0810, 176.0670, 114.0317	Trimethyl citrate	DO
P17	2.637	312.1598	312.1600	-0.6	C_19_H_21_NO_3_	312.1598, 283.1325, 350.1147	Pronuciferine	LR
P18	2.680	623.1976	623.1976	0	C_29_H_36_O_15_	647.1799, 605.1876, 477.1402, 179.0350, 161.0244	Acteoside	CH
P19	2.720	609.1447	609.1456	-1.5	C_27_H_30_O_16_	465.1019,303.1540,287.1847,300.1956,271.6077	Rutin	DO
P20	2.860	479.1554	479.1553	-0.2	C_23_H_28_O_11_	479.1554, 525.1622, 316.0961	Paeoniflorin	PL
M5	2.980	465.1397	465.1397	0.0	C_22_H_26_O_11_	345.0609, 327.0577, 289.1096	Demethylene hydroxyl oxypaeoniflorin	PL
P21	3.592	259.0974	259.0970	1.5	C_15_H_16_O_4_	259.0974, 241.0866, 231.1027, 213.0919	Linderane	LR
M6	3.930	507.1758	507.1749	1.8	C_21_H_32_O_14_	345.1164, 327.1046, 283.1035, 165.1022, 121.1049	Desbenzoyl product of paeoniflorin-O-glucuronide	PL
P22	4.341	269.0456	269.0450	2.2	C_15_H_10_O_5_	269.0456, 251.0356, 315.0505	Genistein	UR
M7	4.520	261.0068	261.0069	-0.4	C_9_H_10_O_7_S	215.0123, 171.0015	3,4-dihydroxyphenylpropionate sulfate	PL
M8	4.560	261.0081	261.0069	4.6	C_9_H_10_O_7_S	261.0081, 242.9960, 224.9858	Dihydroxyphenylpropionate sulfate	LR
P23	4.603	301.0349	301.0348	0.3	C_15_H_10_O_7_	301.0349, 283.0255, 107.0135	Quercetin	LR
P24	4.250	385.2134	385.2127	1.8	C_22_H_28_N_2_O_4_	407.1909,269.1715,160.0771	Rhynchophylline	UR
P25	4.960	429.1172	429.1186	-3.3	C_22_H_22_O_9_	429.1172, 267.0670	Ononin	DO
M9	5.050	199.0970	199.0980	5	C_10_H_14_O_4_	151.1002, 123.0988, 108.0978	Paeonimetabolin I	PL
M10	5.424	389.1057	389.1059	-0.5	C_20_H_22_O_6_S	389.1057, 779.2169	Yakuchinone B-O-sulfate	AO
M11	5.530	449.1440	449.1448	-1.8	C_17_H_24_O_10_	327.1077,309.0975,287.0925,165.0549	Demethylene hydroxyl paeoniflorin	PL
M12	5.620	399.1905	399.1920	-3.8	C_22_H_26_N_2_O_5_	797.3782, 421.1731, 399.1905, 226.1454, 196.0944, 174.0511, 146.0638	Dehydrogenation and hydroxy rhynch-ophylline	UR
M13	5.650	383.0447	383.0437	2.6	C_16_H_15_O_9_S	201.8997, 179.9805, 106.9908	3-O-methylepicatechin-O-sulfate	PL
M14	5.720	285.0756	285.0763	-2.5	C_16_H_12_O_5_	285.0756, 267.0666, 307.0575	Izalpinia	AO
M15	5.758	375.1291	375.1289	-0.5	C_16_H_24_O_10_	375.1291, 345.1186	Desbenzoylpaeoniflorin	PL
M16	6.288	460.1612	460.1608	0.9	C_23_H_27_NO_9_	460.1612, 506.1664, 921.3318	Coclaurine-O-glucuronide	LR
M17	6.560	399.1911	399.1920	-2.3	C_22_H_28_N_2_O_5_	399.1911, 445.1967	Hydroxyrhynchophylline	UR
M18	6.694	491.2295	491.2281	2.8	C_26_H_34_O_9_	491.2295, 473.2177, 455.2087	Oxyphyllacinol-O-glucuronide	AO
P26	7.014	285.0406	285.0399	2.5	C_15_H_10_O_6_	285.0406, 249.0190, 249.0190	Kaempferol	UR
P27	7.392	413.3763	413.3783	-4.8	C_29_H_50_O	413.3763, 443.3904, 371.3318	Sitosterol/isomer	LR
P28	7.410	218.1744	218.1749	-2.3	C_15_H_22_O	203.9254,190.0854,161.5551,147.9437	Nootkatone	AO
M19	7.991	527.0490	527.0496	-1.1	C_21_H_18_O_14_S	527.0490, 1055.1084	Genistein-O-sulfate	UR
M20	7.849	151.0401	151.0395	4	C_8_H_8_O_3_	151.0401, 135.0442, 303.0870	Pyrolysis production of ferulic acid	UR
M21	8.703	493.1714	493.1710	0.8	C_24_H_30_O_11_	493.1714, 987.3504, 415.1223	Methylalbiflorin	PL
P29	8.713	623.1995	623.1976	3	C_29_H_36_O_15_	623.1995, 459.1286, 295.0597	Hydrolysis reaction of echinacoside	CH
M22	9.213	317.1025	317.1018	-2.2	C_17_H_18_O_6_	317.1025, 363.1080, 299.0919	Albiflorinaglycone	PL
M23	9.296	509.1653	509.1659	-1.2	C_24_H_30_O_12_	509.1653, 491.1569, 479.1538	Methyloxyalbiflorin	PL
P30	9.306	283.2645	283.2637	2.8	C_18_H_36_O_2_	283.2645, 329.2692, 269.2475	Octadecanoic acid	CH
M24	9.666	447.0939	447.0927	2.7	C_21_H_18_O_11_	447.0939, 429.0823, 411.0716	Genistein-O-glucuronide	UR
M25	9.670	477.0678	477.0669	1.9	C_21_H_18_O_13_	477.0678, 283.0447, 523.0721	Quercetin-O-glucuronide	LR
P31	10.590	297.0409	297.0399	3.4	C_16_H_10_O_6_	297.409, 343.0437	Irilone	UR
M26	10.846	209.0820	209.0814	2.9	C_11_H_12_O_4_	289.0820, 178.0625, 191.0702	Methylferulic acid	UR
P32	11.253	1073.5540	1073.5532	0.7	C_53_H_86_O_22_	1073.5540, 1119.5554, 749.4459	Macranthoside	UR
P33	12.110	413.3788	413.3783	1.2	C_29_H_48_O	413.3788, 367.3365, 301.2516	Sitosterol/isomer	LR
P34	12.170	749.4482	749.4476	0.8	C_41_H_66_O_12_	751.4638, 795.4532, 603.3890	Kalopanaxsaponin A	UR
P35	12.294	313.1818	313.1804	4.5	C_20_H_26_O_3_	313.1818, 295.1712, 277.1591	Oxyphyllacinol	AO
M27	13.365	521.1659	521.1635	-4.6	C_25_H_30_O_12_	521.1659, 1043.3396	Acetylation paeoniflorin	PL

UR, Uncaria rhynchophylla; CH, Cistanches Herba; PL, Paeonia lactiflora; LR, Linderae Radix; DO, Dioscorea opposite; AO, Alpinia oxyphylla.

### Prototypes constituents and metabolites screening of WSYGD in mouse plasma

### Analysis of prototype constituents

**Figure 7:**
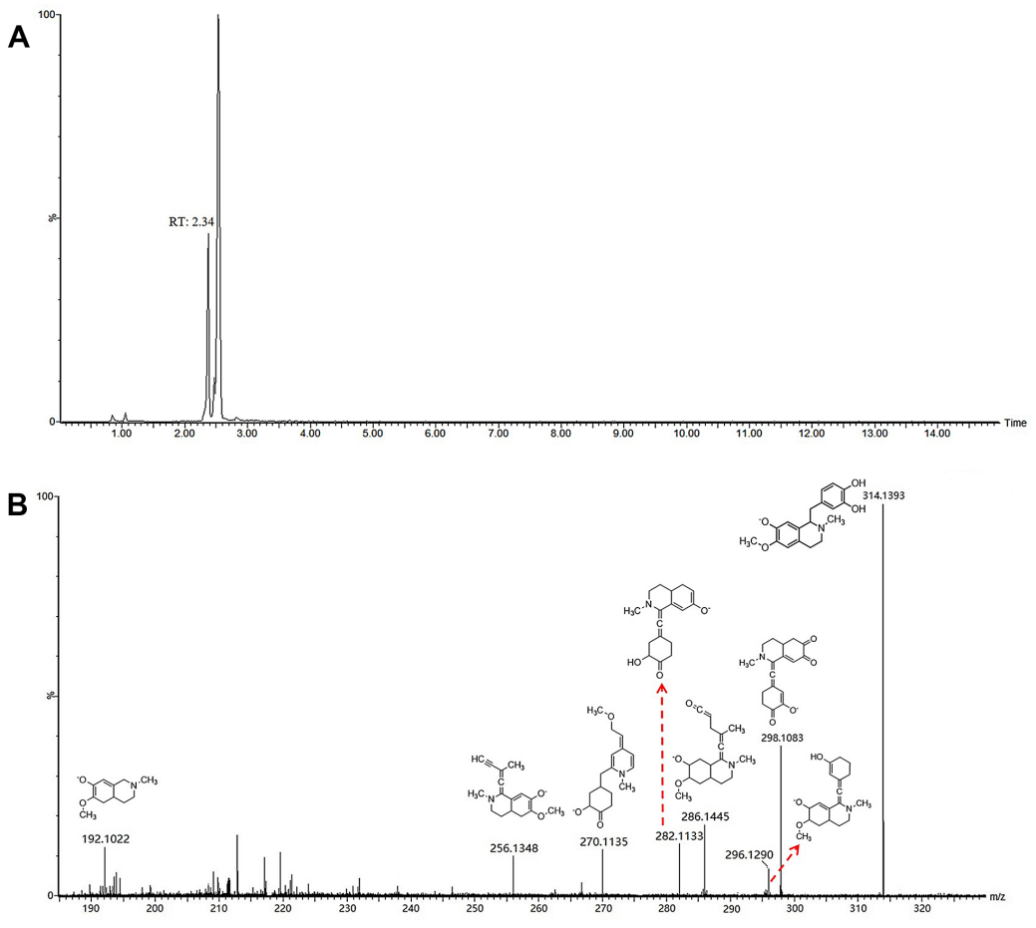
Chromatograms (**A**) and fragmentations and mode assignments (**B**) of coclaurine.

To elucidate the MS/MS information of the exogenous using UPLC-ESI-Q-TOF-MS technique, a comprehensive comprehending of retention regularities and changes in mass spectra of the prototype constituents is essential. Combined the exact mass < 5.0 ppm, the composition of elemental can be explicit acknowledged. By analyzing their accurate mass and MS fragment pathways along with the available literature, 97 prototype ingredients were identified. Taken the compound of P13 as an example, P13 had a molecular ion [M-H] ^–^ at m/z 314.1393 and produce fragmentation with the peak at m/z 298.1083 [M-H-CH_4_]^-^, 286.1445 [M-H-CO]^-^, 282.1133 [M-H-CH_3_-OH]^-^, 270.1135 [M-H-CH_4_-CO]^-^, 256.1348 [M-H-C_2_H_3_O_2_]^-^, 192.1022 [M-H-C_7_H_6_O_2_]^-^. The chemical formula of P13 was conjectured to be C_18_H_21_NO_4_ based on the elemental composition, unsaturation degree and relative abundance. Therefore, ion P13 was deduced preliminarily as Coclaurine (Figure 7).

### Characterization of mouse metabolites of WSYGD

**Figure 8:**
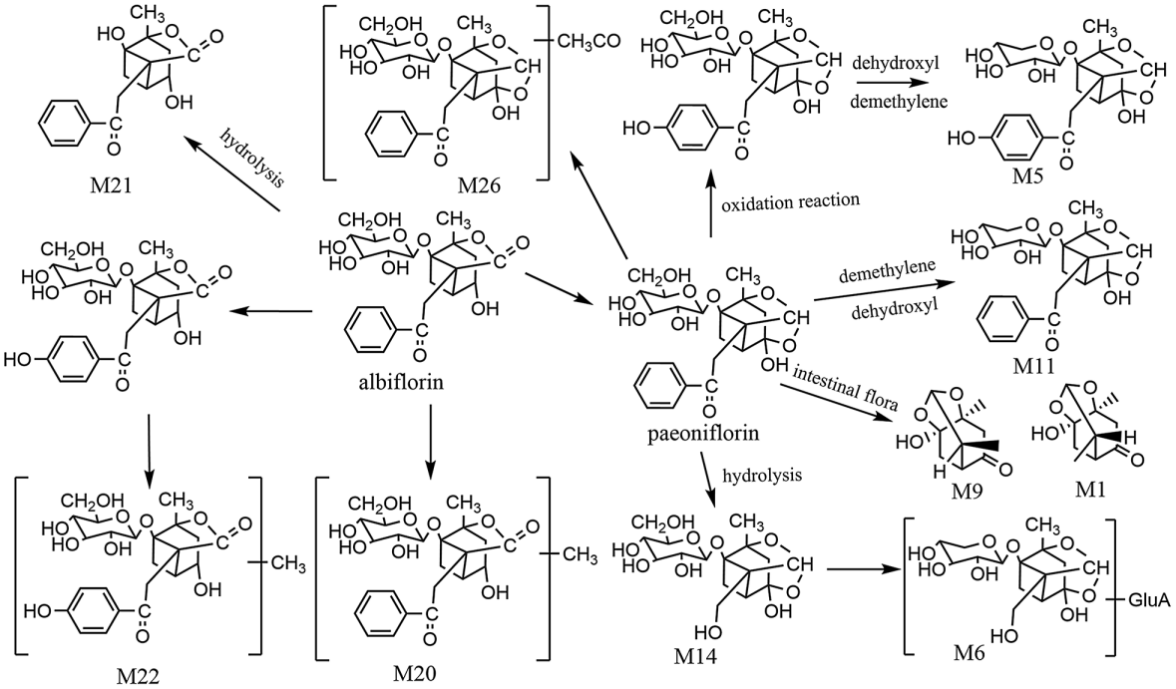
The proposed metabolic profiles of paeoniflorin-related metabolites.

The total ion chromatogram of the endogenous and exogenous compounds in plasma after oral administration of WSYGD was analyzed by the software of Metabolynx 4.1 (Supplementary Figure 2). The possible metabolic pathway of major compounds is indicated in Figure 8. Most of the metabolites remained the skeleton structural characteristics which absorbed in blood [17], the study of fragmentation pathways accelerated the authentication of metabolites from WSYGD in plasma.

The retention time of M9 chromatographic peak was 5.050 min with the [M-H]^-^ ion at m/z 199.0970 and the characterized molecular ion at m/z 151.1002, 123.0988, 108.0978, and the fragmentation behavior was confirmed in accordance with previous literature [18]. Simultaneously, M1 was found on RT 0.402 minute with the ion of [M+H] ^+^ at m/z 201.1120. Therefore, M9 and M1 were identified as Paeonimetabolin I and Paeonimetabolin II.

Metabolite M2 exhibited the ion of [M-H]^-^ at m/z 297.0616 and was obtained at 0.720 minute. The chemical formula of M2 was C_13_H_14_O_8_, which was 176 Da higher than that of benzoic acid and exhibited the fragmentation of 121.0132 [M-GluA] ^+^. M2 was tentatively assigned as glucuronidated product of Benzoic acid-O-glucuronide.

Metabolites M14 (t_R_=1.35 min) showed the ion of [M-H]^-^ at m/z 375.1291, which the molecular formula was calculated to be C_16_H_24_O_10_. The fragment ion which produced by [M-H]^-^ was exhibited at 375.1291 [M-C_7_H_4_O]^-^. Simultaneously, it was 104 Da lower than paeoniflorin, and the characterized fragment ion at m/z 345.1186, 327.1124, 283.1115. Ultimately, M14 [19] was tentatively identified as desbenzoylpaeoniflorin. Metabolite M6 was detected on the Rt 2.51 min of chromatogram and produced an ion of [M-H]^-^ at m/z 507.1758 (C_21_H_32_O_14_), which was 132 Da (C_6_H_8_O_6_-CO_2_) higher than desbenzoylpaeoniflorin, indicating that the ingredients were conjugated with glucuronic acid, happening phase II in metabolic processes. Simultaneously, the main products ion of 327.1046, 283.1035, 165.1022, 121.1049 was present based on the fragmentation pattern. Therefore, M6 was designated as desbenzoyl product of paeoniflorin-O-glucuronide. Metabolite M11(t_R_=5.53 min) presented an ion of [M-H]^-^ at m/z 449.1440 (C_17_H_24_O_10_). Therefore, M5 and M11 were designated as demethylene hydroxyl oxypaeoniflorin, demethylene hydroxyl paeoniflorin. M26 exhibited quasi-molecular [M-H]^-^ ion at m/z 521.1659 and the chemical formula calculated to be C_25_H_30_O_12_, which was 42 Da lower than paeoniflorin. M26 was identified as acetylation paeoniflorin.

Paeoniflorin was easy isomerize into albiflorin, and the intensity of the peaks is different. M20 had an [M-H]^-^ ion at m/z 493.1714 (C_24_H_30_O_11_), which was 15 Da higher than albiflorin. M22 emerged an ion of [M-H]^-^ at m/z 317.1025(C_17_H_18_O_6_) and 30 Da more than albiflorin. Therefore, M20 and M22 were identified as methylalbiflorin and methyloxyalbiflorin. Metabolite M21 exhibited the ion of [M-H]^-^ at m/z 317.1025 (C_17_H_18_O_6_), which was 162 Da higher than that of albiflorin. It was indicating that the constituent was obtained through the loss of glycoside group. M21 was designated as albiflorinaglycone.

M11(t_R_ 5.62 min) and M16 (t_R_ 6.56 min) emerged the ions of [M+H]^+^ at m/z 399.1950 and 399.1911, respectively. Simultaneously, the chemical of M11 and M16 have the same molecular formula, C_22_H_26_N_2_O_5_, and were 16 Da more than that of isocorynoxeine. M11 and M16 were acknowledged as isocorynoxeine-N-oxide and corynoxeine-N-oxide based on the reserve time, exact mass and diagnostic fragment ions [20]. The other metabolites, such as M2, M3, M4, M7, M8, M10, M12, M13, M15, M17, M18, M19, M23, M24, M25, were identified using the same methods.

### Quantification of ten main constituents in WSYGD

**Figure 9:**
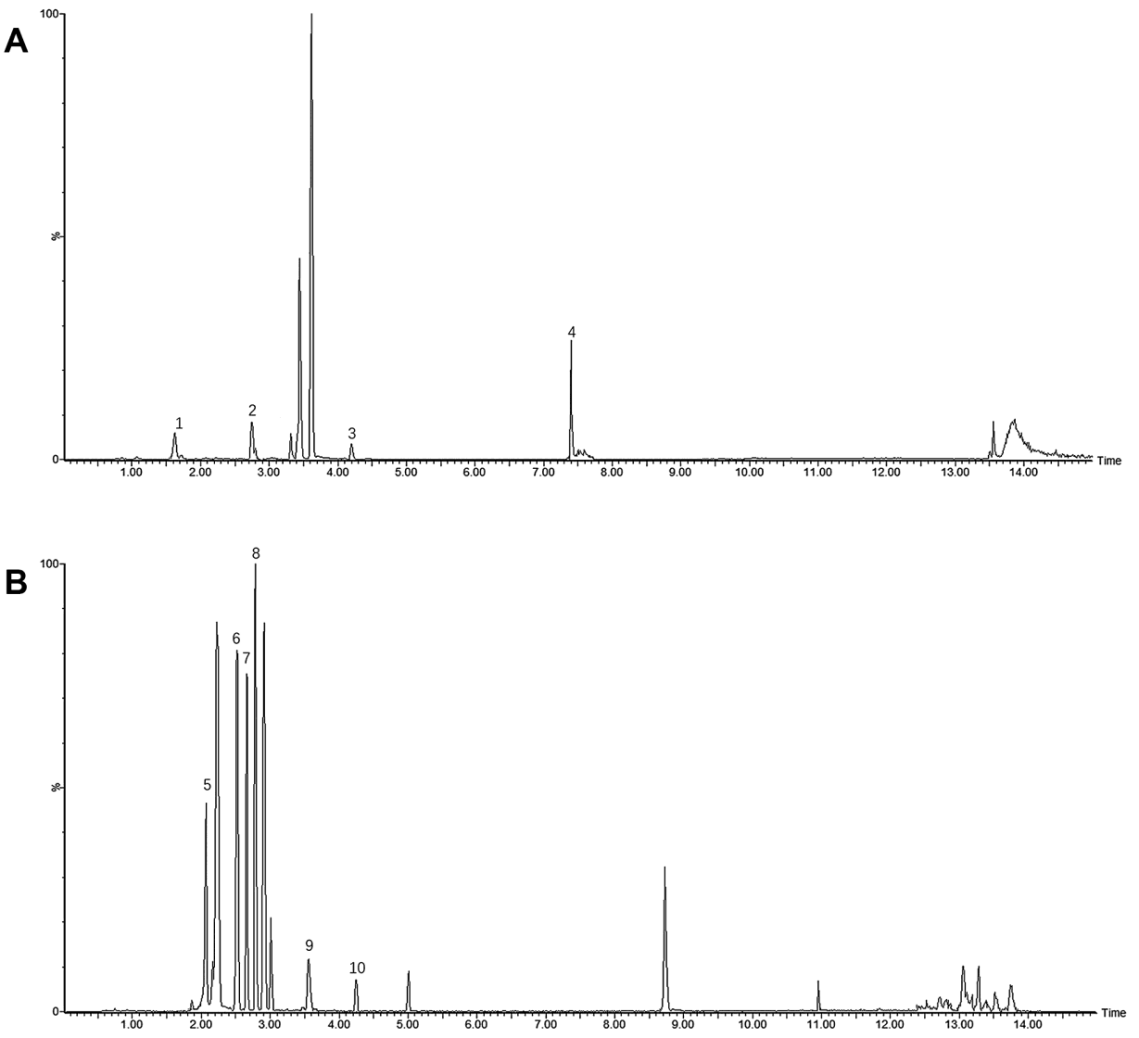
UHPLC-MS/MS spectra and their structures of ten mixture standard compounds in positive mode (**A**) (1 Geniposidic acid; 2 Coclaurine; 3 Rhynchophylline; 4 Nootkatone) and negative mode (**B**) (5 Rutin; 6 Echinacoside; 7 Acteoside; 8 Paeoniflorin; 9 Linderane; 10 Quercetin).

The correlation coefficient (r^2^) of the calibration curve > 0.999, indicating good linearity. The LOD, LOQ, intra- and inter day precision, repeatability, and recoveries were tested and the obtained results were summarized in Tables 2, 3, demonstrating good repeatability for the simultaneous quantification of the ten main constituents in WSYGD. Their typical chromatograms were shown in Figure 9.

**Table 2:** Statistical results of linear regression equation analysis in the determination of main compounds.

Analyte	Regression equation	r^2^	LOD(μg/mL)	LOQ(μg/mL)	Linear range(μg/mL)	content(μg/g)
Geniposidic acid	Y=530.4614X+560.4806	0.9996	0.082	0.280	25.67-256.7	35.45
Coclaurine	Y=497.2478X-286.269	0.9993	0.095	0.225	19.97-199.7	26.08
Rhynchophylline	Y=98966.3056X+132771.6067	0.9999	0.065	0.122	1.72-172	23.56
Nootkatone	Y=59913.3219X+160.8017	0.9992	0.115	0.378	15.25-152.5	18.93
Rutin	Y=3 200.4089 X+99.2290	0.9994	0.078	0.215	27.2-870	120.35
Echinacoside	Y=136.6601X-93.5573	0.9996	0.085	0.189	11.97-119.7	45.21
Acteoside	Y=25839.1X-1485.23	0.9999	0.060	0.120	25.56-255.6	56.50
Paeoniflorin	Y=321.0675X+135.5458	0.9998	0.065	0.546	26.78-267.8	98.58
Linderane	Y=3269.2578X+4578.7369	0.9999	0.108	0.376	1.64-16.4	3.67
Quercetin	Y=5 365.7657X-7.6742	0.9999	0.105	0.256	1.02-15.2	1.23

**Table 3:** Precision, repeatability, and stability data of ten compounds of WSYGD.

**Analyte**	**Precision**				**Repeatability**		**Recovery**	
	**Intraday(n=6)**		**Interday(n=6)**		**(n=6)**		**(n=6)**	
	**Mean(μg/g)**	**RSD(%)**	**Mean(μg/g)**	**RSD(%)**	**Mean(μg/g)**	**RSD(%)**	**Mean(%)**	**RSD(%)**
Geniposidic acid	37.25	2.52	36.13	1.85	33.38	2.34	98.98	1.87
Coclaurine	25.57	1.56	25.12	2.26	27.13	2.78	102.58	2.90
Rhynchophylline	23.76	2.03	25.30	1.80	26.09	1.86	99.07	1.84
Nootkatone	18.58	2.05	19.09	2.56	18.39	2.68	104.10	1.76
Rutin	120.34	1.97	123.47	2.80	120.56	2.98	102.67	1.08
Echinacoside	45.89	2.65	46.27	1.85	46.07	1.24	100.05	2.37
Acteoside	55.13	2.59	57.09	2.58	55.28	2.83	102.18	2.87
Paeoniflorin	98.03	1.82	99.02	2.49	98.02	1.06	98.05	1.38
Linderane	3.68	2.97	3.98	1.09	3.12	1.96	103.08	2.09
Quercetin	1.24	2.01	1.12	2.78	1.29	2.03	101.50	2.37

## CONCLUSIONS

Parkinson disease, the common disabling neurodegenerative disease, was bound up with the loss of dopaminergic neurons in the SN. Proinflammatory mediators was released caused by the microglia activates which associated with injured dopaminergic neurons. Ultimately, it was caused the successive dopaminergic neural degeneration correlative with the aggregated proteins, such as nitrated α-syn, of Parkinson disease released. However, it is debatable whether or not WSYGD could make any difference. According to this research, (1) WSYGD could ameliorate the PD behaviors of rotenone-intoxicated mice, down-regulate the expression of α-synuclein and refrain from the reduce of SN dopaminergic neurons; (2) the mice of rotenone-intoxicated had neuroinflammatory symptoms such as raised conveyance of peripheral immune factors, which could be improved by WSYGD. Simultaneously, we developed a dependable method to discriminate the diverse the endogenous and exogenous compounds originate from complicated herbal extracts. Recently, LC-MS/MS, due to its high selectivity, sensitivity and chromatographic resolution, has been utilized for the authentication and quantitation of bioanalysis of complex matrix samples. Therefore, the combine of LC-MS/MS and multivariate statistical analysis was built to research the chemical ingredient and xenobiotics in mice after oral administration of WSYGD. By this means, a total of 97 chemical constituents and 62 metabolites were characterized tentatively. More importantly, 35 prototype constituents and 27 metabolites of WSYGD after oral administration were screened based on the fragmentation behavior of WSYGD in mouse plasma. Simultaneously, quantitative analysis of ten main constituents in WSYGD was replicated. It is still necessary to make further efforts to the biological evaluation and the concrete the characteristics of drug metabolism among the multicomponent. The results will supply one species of rapid and highly efficient method for the authentication of ingredients and the obtained knowledge that might be helpful to guide the clinical applications of these TCM in PD.

## Supplementary Material

Supplementary Figures

Supplementary Table 1
