# Supplementary material for: UHPLC-MS-based metabolomics and chemoinformatics study reveals the neuroprotective effect and chemical characteristic in Parkinson’s disease mice after oral administration of Wen-Shen-Yang-Gan decoction
Source: Aging (Albany NY). 2021 Aug 2;13(15):19510–28. doi: 10.18632/aging.203361 (PMC8386550; doi:10.18632/aging.203361)
Supplement: Supplementary Figures [file aging-13-203361-s001.pdf]

SUPPLEMENTARY FIGURES

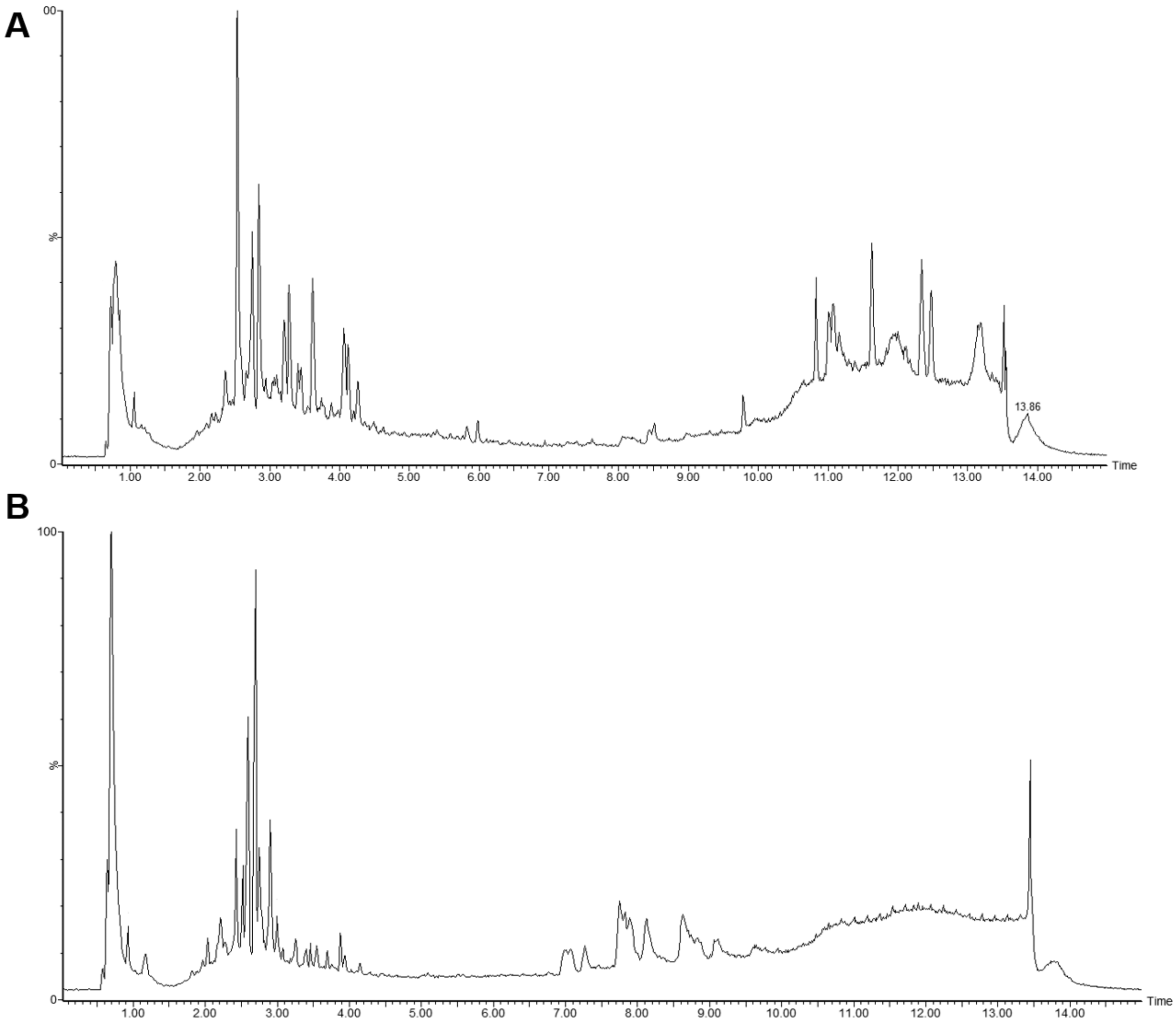

Supplementary Figure 1. The total ion chromatogram of WSYGD extract in positive ion (**A**) and negative ion (**B**).

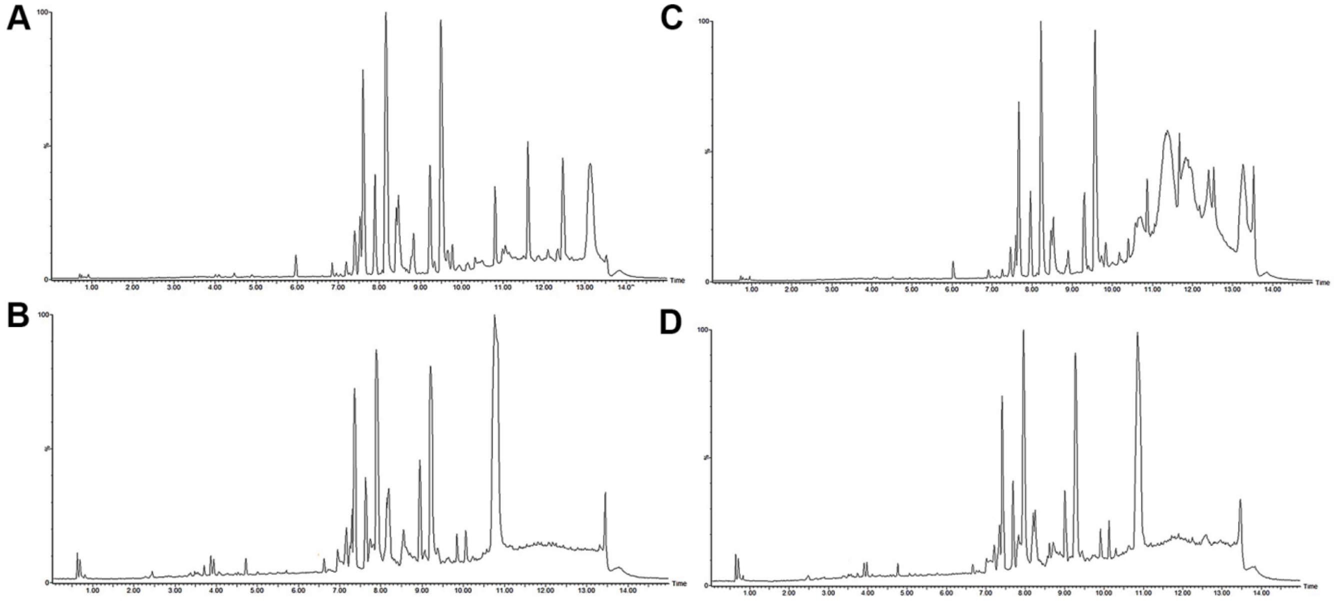

Supplementary Figure 2. Chromatograms of WSYGD in mouse plasma by UPLC-ESI-Q-TOF MS: (**A**) total ion chromatogram in positive ion mode of blank, (**B**) total ion chromatogram in negative ion mode of blank, (**C**) total ion chromatogram in positive ion mode of WSYGD, (**D**) total ion chromatogram in negative ion mode of WSYGD.
