# Supplementary material for: UHPLC-MS-based metabolomics and chemoinformatics study reveals the neuroprotective effect and chemical characteristic in Parkinson’s disease mice after oral administration of Wen-Shen-Yang-Gan decoction
Source: Aging (Albany NY). 2021 Aug 2;13(15):19510–28. doi: 10.18632/aging.203361 (PMC8386550; doi:10.18632/aging.203361)
Supplement: Supplementary Table 1 [file aging-13-203361-s002.docx]

SUPPLEMENTARY TABLE

Supplemenatry Table 1. Components identified from Wen-Shen-Yang-Gan decoction.

| **No.** | **t_R_ (min)** | **Detected mass [M-H]^-^/[M+H]^+^** | **Theoretical exact mass (Da)** | **Mass error (ppm)** | **Molecular formula** | **MS/MS (m/z)** | **Assigned identity** | **Source** |
| --- | --- | --- | --- | --- | --- | --- | --- | --- |
| **1** | 0.67 | 191.0551 | 191.0556 | -2.6 | C_7_H_12_O_6_ | 173.0459,111.0447 | Quinic acid | PL |
| **2** | 0.80 | 118.0871 | 118.0868 | 2.5 | C_5_H_11_NO_2_ | 118.0871,74.0972 | Glycine betaine | CH |
| **3** | 1.06 | 125.0240 | 125.0239 | 0.8 | C_6_H_6_O_3_ | 165.9513, 107.0133, 95.0125, 92.9979 | 5- hydroxymethyl furfural | AO |
| **4** | 1.13 | 199.0605 | 199.0606 | -0.5 | C_9_H_10_O_5_ | 181.0137, 169.0501, 155.0708, 151.0395 | Syringic acid | UR |
| **5** | 1.85 | 311.1658 | 311.1647 | 3.5 | C_20_H_22_O_3_ | 311.1658, 174.1568, 136.1689 | Yakuchinone B | AO |
| **6** | 1.87 | 151.0604 | 151.0606 | -1.3 | C_5_H_12_O_5_ | 175.026, 133.0501, 91.0395, 89.0239, 61.0290, 59.0133 | D-Arabitol | DO |
| **7** | 1.91 | 487.1436 | 487.1452 | -3.3 | C_21_H_28_O_13_ | 511.1199 | Cistanoside F | CH |
| **8** | 1.93 | 355.1013 | 355.1029 | -4.5 | C_16_H_18_O_9_ | 355.1015,127.1123 | Chlorogenic acid | UR |
| **9** | 1.96 | 1.9600 | 375.1295 | 1.1 | C_16_H_24_O_10_ | 399.9706,215.5983 | Adoxosidic acid | CH |
| **10** | 1.98 | 387.1084 | 387.1080 | 1.0 | C_20_H_18_O_8_ | 795.159, 316.1589, 128.1576 | Cleomiscosin B | UR |
| **11** | 2.02 | 415.1023 | 415.1029 | -1.4 | C_21_H_20_O_9_ | 415.1018,399.1080, 253.0501 | Daidzin | DO |
| **12** | 2.03 | 341.1251 | 341.1236 | 4.4 | C_16_H_22_O_8_ | 343.1408,165.0922,325.1274 | Coniferin | CH |
| **13** | 2.06 | 373.1145 | 373.1135 | 2.7 | C_16_H_22_O_10_ | 375.1284, 313.1287, 213.0727, 179.0708, 121.0653 | Geniposidic acid | CH |
| **14** | 2.11 | 371.1334 | 371.1334 | -2.2 | C_17_H_22_O_9_ | 393.4806 | Sinapaldhyde glucoside | CH |
| **15** | 2.15 | 375.1286 | 375.1291 | -1.3 | C_16_H_24_O_10_ | 345.1175,213.0749,165.0205 | Desbenzoylpaeoniflorin | PL |
| **16** | 2.18 | 385.2136 | 385.2127 | 2.0 | C_22_H_28_N_2_O_4_ | 407.1945,241.1411,187.0815 | Isorhynchophylline | UR |
| **17** | 2.20 | 314.1393 | 314.1392 | 0.3 | C_18_H_21_NO_4_ | 314.1389, 298.1134, 282.0973, 256.0876, 192.1030 | Coclaurine | LR |
| **18** | 2.25 | 577.1348 | 577.1346 | 0.3 | C_30_H_26_O_12_ | 577.1325,287.0614,559.1246 | Proanthocyanidins | LR |
| **19** | 2.27 | 369.1180 | 369.1186 | -1.6 | C_17_H_20_O_9_ | 333.0968,259.0818, 173.0450, 163.0395 | Methyl chlorogenic acid | DO |
| **20** | 2.31 | 328.1550 | 328.1549 | 0.3 | C_19_H_21_NO_4_ | 311.0546,296.0355,280.0847, 251.1239,222.0667 | Scoulerin | LR |
| **21** | 2.33 | 495.1496 | 495.1503 | -1.4 | C_23_H_28_O_12_ | 419.0968,333.0974,137.0247 | Oxypaeoniflorin | PL |
| **22** | 2.37 | 863.1824 | 863.1823 | 0.1 | C_45_H_36_O_18_ | 575.6221,431.1913,287.0521 | Cinnamtannin B1 | LR |
| **23** | 2.42 | 479.3027 | 479.3009 | 3.8 | C_27_H_44_O_7_ | 479.3014, 461.2909, 345.2071, 319.1915, 301.1809 | Crustecdysone | DO |
| **24** | 2.49 | 329.0653 | 329.0661 | -2.4 | C_17_H_14_O_7_ | 329.0654,375.0728 | Tricin | UR |
| **25** | 2.56 | 785.2506 | 785.2504 | 0.3 | C_35_H_46_O_20_ | 809.2402 | Echinacoside | CH |
| **26** | 2.59 | 169.0506 | 169.0501 | 3.0 | C_8_H_8_O_4_ | 152.0623,108.9665,82.9359 | Vanillic acid | CH |
| **27** | 2.61 | 799.2659 | 799.2661 | -0.3 | C_36_H_48_O_20_ | 823.2540,839.3042 | Cistanoside A | CH |
| **28** | 2.65 | 257.0820 | 257.0814 | 2.3 | C_15_H_12_O_4_ | 179.0773,152.2337,124.9100 | Pinocembrin | LR |
| **29** | 2.66 | 653.6218 | 653.6237 | -2.9 | C_45_H_80_O_2_ | 396.243, 353.3203, 313.2526, 295.2420, 95.0855 | Stigmasterol palmiate | AO |
| **30** | 2.67 | 312.1598 | 312.1600 | -0.6 | C_19_H_21_NO_3_ | 312.1598,283.1325,350.1147 | Pronuciferine | LR |
| **31** | 2.68 | 301.0710 | 301.0712 | -0.7 | C_16_H_12_O_6_ | 288.0591,287.0548,260.0604 | 3'-Methoxy-4',5,7-trihydroxyflavone | UR |
| **32** | 2.70 | 330.1701 | 330.1705 | -1.2 | C_19_H_23_NO_4_ | 299.1263,287.0580,267.1021 | reticuline | LR |
| **33** | 2.72 | 609.1447 | 609.1456 | -1.5 | C_27_H_30_O_16_ | 465.1019,303.1540,287.1847,300.1956,271.6077 | Rutin | DO |
| **34** | 2.74 | 359.1344 | 359.1342 | 0.6 | C_16_H_22_O_9_ | 381.1304 | Sweroside | DO |
| **35** | 2.77 | 195.0660 | 195.0657 | 1.5 | C_10_H_10_O_4_ | 217.1205, 163.0395, 135.0446 | Methyl caffeic acid | DO |
| **36** | 2.78 | 623.1976 | 623.1976 | 0.0 | C_29_H_36_O_15_ | 647.1799, 605.1876, 477.1402, 179.0350, 161.0244 | Isoacteoside | CH |
| **37** | 2.79 | 623.1969 | 623.1973 | -0.5 | C_29_H_36_O_15_ | 605.1870, 487.1452, 477.1397, 179.0344, 161.0239 | Acteoside | CH |
| **38** | 2.80 | 623.1973 | 623.1976 | -0.5 | C_29_H_36_O_15_ | 689.1713, 647.1909 | Verbascoside/isomer | CH |
| **39** | 2.79 | 827.2628 | 827.2610 | 2.2 | C_37_H_48_O_21_ | 851.1840 | Tubuloside A | CH |
| **40** | 2.81 | 163.0397 | 163.0395 | 1.2 | C_9_H_8_O_3_ | 109.0297,101.0246 | cis-4-coumaric acid | LR |
| **41** | 2.82 | 827.2628 | 827.2610 | 2.2 | C_34_H_44_O_19_ | 779.6537 | Arenarioside | CH |
| **42** | 2.84 | 593.1508 | 593.1506 | 0.3 | C_27_H_30_O_15_ | 617.001,575.1401, 285.0399, 163.0606, 145.0501 | Biorobin | DO |
| **43** | 2.85 | 235.0823 | 235.0818 | 2.1 | C_9_H_14_O_7_ | 235.0810,176.0670,114.0317 | Trimethyl citrate | DO |
| **44** | 2.86 | 179.0713 | 179.0708 | 2.8 | C_10_H_10_O_3_ | 147.1200,119.0876 | 4-Hydroxycinnamic acid methyl ester | UR |
| **45** | 2.96 | 277.1079 | 277.1076 | 1.1 | C_15_H_18_O_5_ | 261.1095,243.8917,225.9301,215.8739 | Linderolide A | LR |
| **46** | 3.04 | 479.1552 | 479.1553 | -0.2 | C_23_H_28_O_11_ | 525.1629，327.1075, 165.0206, 121.0286 | Paeoniflorin | PL |
| **47** | 3.05 | 479.1552 | 479.1553 | -0.2 | C_23_H_28_O_11_ | 525.1629,435.1658,357.1177,121.0287 | Albiflorin | PL |
| **48** | 3.12 | 263.1228 | 263.1283 | 1.9 | C_15_H_20_O_4_ | 247.1209,229.0556,219.8449,201.1391 | Linderolide K | LR |
| **49** | 3.15 | 342.1700 | 342.1705 | -1.5 | C_20_H_23_NO_4_ | 311.1273,296.1035,280.1088,232.1392,86.7216 | N- Methyl scoulerin | LR |
| **50** | 3.17 | 447.0948 | 447.0927 | 4.5 | C_21_H_20_O_11_ | 431.0978, 287.124,269.0450, 257.0450, 163.0606 | Cynaroside | UR |
| **51** | 3.19 | 543.2332 | 543.2343 | -2.0 | C_29_H_34_N_2_O_10_ | 580.1499, 364.1503 | Cadambine | UR |
| **52** | 3.26 | 637.2111 | 637.2132 | -3.3 | C_30_H_38_O_15_ | 661.2089 | Cistanoside C | CH |
| **53** | 3.28 | 369.1820 | 369.1814 | 1.6 | C_21_H_24_N_2_O_4_ | 223.0688,208.1036,180.0802,130.0098 | Uncarine E | UR |
| **54** | 3.29 | 623.1979 | 623.1976 | 0.5 | C_29_H_36_O_15_ | 477.1400 | verbascoside/isomer | CH |
| **55** | 3.86 | 195.0665 | 195.0657 | 4.1 | C_10_H_10_O_4_ | 151.1123,119.0858 | Ferulic acid | UR |
| **56** | 3.42 | 631.1657 | 631.1663 | -1.0 | C_30_H_32_O_15_ | 613.1528,169.0129,313.0555.465.1363,479.1113,491.1175 | Galloyl oxypaeoniflorin | PL |
| **57** | 3.49 | 259.0969 | 259.0970 | -0.4 | C_15_H_16_O_4_ | 243.0658,225.9225,197.8069,187.9256 | Linderolide D | LR |
| **58** | 3.53 | 259.0969 | 259.0970 | -0.4 | C_15_H_16_O_4_ | 259.0974,241.0866,231.1027,213.0919 | Linderane | LR |
| **59** | 3.65 | 330.1704 | 330.1705 | -0.3 | C_19_H_23_NO_4_ | 299.1263,192.1023,175.0761 | Reticulline | LR |
| **60** | 3.95 | 218.1590 | 218.1592 | -0.9 | C_15_H_20_O | 201.7407,187.0766,173.5310,159.8545 | Dehydronootkatone | AO |
| **61** | 4.04 | 527.1404 | 527.1401 | 0.6 | C_23_H_28_O_14_ | 497.1254,479.1164,365.0847,271.0563 | 6′-O-galloyl desbenzoylpaeoniflorin | PL |
| **62** | 4.11 | 419.2413 | 419.2434 | -5.0 | C_24_H_34_O_6_ | 324.2399, 305.2425 | Desacetylcinobufotalin | DO |
| **63** | 4.13 | 243.1024 | 243.1021 | 1.2 | C_15_H_16_O_3_ | 227.9875,217.8600,199.1121,181.0670 | Linderalactone | LR |
| **64** | 4.16 | 261.1126 | 261.1127 | -0.4 | C_15_H_18_O_4_ | 245.1081,217.8583,199.1127,181.9156 | Linderolide C | LR |
| **65** | 4.22 | 499.2089 | 499.2080 | 1.8 | C_26_H_30_N_2_O_8_ | 521.2217, 471.2216, 320.2199, | Vincosamide | UR |
| **66** | 4.25 | 385.2134 | 385.2127 | 1.8 | C_22_H_28_N_2_O_4_ | 407.1945,269.1705,160.0762 | Rhyncholphylline | UR |
| **67** | 4.29 | 369.2173 | 369.2178 | -1.4 | C_22_H_28_N_2_O_3_ | 337.1914, 238.1441, 226.1440, 194.1177, 110.0963 | Hirsutine | UR |
| **68** | 4.32 | 269.0461 | 269.0450 | 4.1 | C_15_H_10_O_5_ | 269.0456,251.0356,315.0505 | Genistein | UR |
| **69** | 4.40 | 301.0332 | 301.0348 | -5.3 | C_15_H_10_O_7_ | 301.0349,283.0255,107.0135 | Quercetin | LR |
| **70** | 4.80 | 261.1128 | 261.1127 | 0.4 | C_15_H_16_O_4_ | 283.1496,543.2967 | Pseudoneoliderane | LR |
| **71** | 4.86 | 583.1826 | 583.1816 | 1.7 | C_30_H_32_O_12_ | 629.1886,553.1705,479.1266,431.1319 | Benzoylalbiflorin | PL |
| **72** | 5.08 | 429.1174 | 429.1186 | -2.8 | C_22_H_22_O_9_ | 429.1172,267.0670, 237.0552 | Ononin | DO |
| **73** | 5.41 | 255.0653 | 255.0657 | -1.6 | C_15_H_10_O_4_ | 253.0811,161.1327 | Daidzein | UR |
| **74** | 5.54 | 235.1695 | 235.1698 | -1.3 | C_15_H_22_O_2_ | 216.6192,201.9004 | 7-epi-teucrenone | AO |
| **75** | 5.58 | 215.1438 | 215.1436 | 0.9 | C_15_H_18_O | 197.8926,187.9238,182.9631,174.9171 | Lindenen | LR |
| **76** | 5.64 | 285.0770 | 285.0763 | 2.5 | C_16_H_12_O_5_ | 307.0785, 285.0763, 255.0657 | Physcion | DO |
| **77** | 5.67 | 315.2331 | 315.2324 | 2.2 | C_21_H_32_O_2_ | 315.2324, 297.2218, 273.2215, 253.1956 | Pregnenolone | DO |
| **78** | 5.83 | 285.0767 | 285.0763 | 1.4 | C_16_H_12_O_5_ | 285.0767 | izalpinia | AO |
| **79** | 7.11 | 269.0804 | 269.0814 | -3.7 | C_16_H_12_O_4_ | 239.1164,225.3016,166.6449,138.5797 | Tectochrysin | AO |
| **80** | 7.12 | 136.0397 | 136.0399 | -1.5 | C_7_H_7_NO_2_ | 94.6656,92.0420,78.0018,65.9941 | Trigonelline | UR |
| **81** | 7.25 | 285.0400 | 285.0399 | 0.4 | C_15_H_10_O_6_ | 285.0406,249.0190,249.0190 | Kaempferol | UR |
| **82** | 7.33 | 118.0191 | 118.0188 | 2.6 | C_4_H_6_O_4_ | 119.0344,141.0171,136.0615 | Succnic acid | AO |
| **83** | 7.41 | 218.1744 | 218.1749 | -2.3 | C_15_H_22_O | 203.9254,190.0854,161.5551,147.9437 | Nootkatone | AO |
| **84** | 7.52 | 413.3777 | 413.3783 | -1.5 | C_29_H_50_O | 413.3763,443.3904,371.3318 | Sitosterol/isomer | CH |
| **85** | 7.86 | 371.1341 | 371.1342 | -0.3 | C_17_H_24_O_9_ | 395.1446, 373.1453, 211.1965 | Syringin | CH |
| **86** | 7.94 | 181.0496 | 181.0501 | -2.8 | C_9_H_8_O_4_ | 163.0395, 151.0387, 135.0446, 105.0340, 71.0133 | Caffeic acid | UR |
| **87** | 8.58 | 279.1588 | 279.1956 | -2.9 | C_16_H_22_O_4_ | 149.0972,223.1279,205.0891 | Dibutyl phthalate | UR |
| **88** | 8.59 | 465.1016 | 465.1033 | -3.7 | C_21_H_22_O_12_ | 445.0771, 299.0192, 283.0243, 179.0556, 161.0450 | Quercetin 3-O-galactoside | UR |
| **89** | 10.58 | 297.0408 | 297.0399 | 3.0 | C_16_H_10_O_6_ | 297.409,343.0437 | irilone | UR |
| **90** | 11.03 | 457.4024 | 457.4046 | -3.1 | C_31_H_52_O_2_ | 415.3940, 397.3834, 357.3521, 99.1174 | β-Sitosterol acetate | DO |
| **91** | 11.42 | 1073.5537 | 1073.5532 | 0.0 | C_53_H_86_O_22_ | 1073.5540,1119.5554,749.4459 | Macranthoside | UR |
| **92** | 11.46 | 449.1088 | 449.1084 | 0.9 | C_21_H_20_O_11_ | 471.1038, 303.0505, 285.0399, 273.0386 | Vincetoxicoside B | DO |
| **93** | 11.84 | 283.2633 | 283.2637 | -1.4 | C_18_H_36_O_2_ | 270.8729 | Octadecanoic acid | CH |
| **94** | 12.10 | 751.4625 | 751.4633 | -1.1 | C_41_H_66_O_12_ | 751.4638,795.4532,603.3890 | Kalopanaxsaponin A | UR |
| **95** | 12.22 | 313.1800 | 313.1804 | -1.3 | C_20_H_26_O_3_ | 313.1818,295.1712,277.1591 | Oxyphyllacinol | AO |
| **96** | 12.35 | 413.3794 | 413.3783 | 2.7 | C_29_H_50_O | 413.3788,367.3365,301.2516 | Sitosterol/isomer | LR |
| **97** | 12.58 | 285.0762 | 285.0763 | -0.4 | C_16_H_12_O_5_ | 285.0760,307.0574,323.0312 | Maackiain/Prunetin | UR |

UR: Uncaria rhynchophylla; CH: Cistanches Herba; PL： Paeonia lactiflora; LR：Linderae Radix; DO: Dioscorea opposite; AO: Alpinia oxyphylla
